# *In vivo* visualization of redox status by high-resolution whole body magnetic resonance imaging using nitroxide radicals

**DOI:** 10.3164/jcbn.18-18

**Published:** 2018-07-25

**Authors:** Tetsuro Uchida, Hitoshi Togashi, Yoshinori Kuroda, Kazuyuki Haga, Mitsuaki Sadahiro, Takamasa Kayama

**Affiliations:** 1 Second Department of Surgery, Faculty of Medicine, Yamagata University, 2-2-2 Iida-Nishi, Yamagata 990-9585, Japan; 2 Health Administration Center, Yamagata University, 2-2-2 Iida-Nishi, Yamagata 990-9585, Japan; 3 Radiation Department, Yamagata University Hospital, 2-2-2 Iida-Nishi, Yamagata 990-9585, Japan; 4 Department of Advanced Cancer Science, Faculty of Medicine, Yamagata University, 2-2-2 Iida-Nishi, Yamagata 990-9585, Japan

**Keywords:** magnetic resonance imaging, redox status, nitroxide radicals

## Abstract

Various diseases are known to be associated with an imbalance of the redox state, but *in vivo* detection of free radicals is difficult. The purpose of this study is to establish a method for *in vivo* visualization of redox status by high-resolution whole-body MRI using nitroxide radicals. A redox-sensitive nitroxide probe, 3-carbamoyl-2,2,5,5-tetramethylpyrrolidine-1-oxyl (carbamoyl-PROXYL), was administered to rats intravenously, and *in vivo* T1-weighted MRI was performed to virtually visualize the redox status of various organs. In experiments using phantoms, a linear relationship between the MRI signal and the carbamoyl-PROXYL concentration persisted up to 80 mM. Among the phantoms, a sample containing 1 mM carbamoyl-PROXYL was readily identifiable. After intravenous injection of carbamoyl-PROXYL, whole-body T1-weighted MRI of the rat provided clear images with good spatial and temporal resolution. The signal intensities of four selected organs (heart, liver, kidney, and intestine) were analyzed quantitatively. The carbamoyl-PROXYL signal peaked and gradually declined due to reduction after intravenous injection. Among the four organs, the organ-specific reduction rate of carbamoyl-PROXYL was highest in the heart, followed by (in order) the liver, kidney, and intestine, and statistical analysis showed that the inter-organ differences were significant. In conclusion, T1-weighted carbamoyl-PROXYL-enhanced MRI provides excellent spatial and temporal imaging of carbamoyl-PROXYL distribution. Furthermore, it provides important functional information pertaining to blood flow and tissue redox activity in individual organs. MRI in combination with carbamoyl-PROXYL has potential clinical application for evaluation of redox activity in whole organs.

## Introduction

**Fig. 1:**
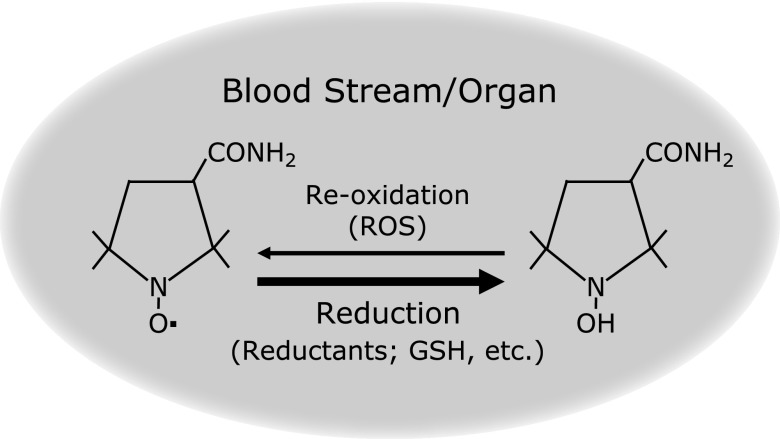
Immediately after administration, carbamoyl-PROXYL is reduced to the corresponding hydroxylamine, losing its characteristics as a free radical. The reduction takes place in the bloodstream and organs. Sulfhydryl (SH) compounds including reduced glutathione are implicated in the process of carbamoyl-PROXYL reduction.

Numerous factors can influence the redox state, either by increasing the rate of production of reactive oxygen species (ROS) or by decreasing antioxidant capacity. An imbalance between ROS and antioxidant defense capability causes oxidative stress, which results in oxidative damage. These reactions are known to be associated with a number of pathological conditions such as atherosclerosis, ischemia-reperfusion, hypertension, diabetes mellitus, brain disorders and neoplastic diseases. It is virtually impossible to directly detect free radicals *in vivo*, but the redox state of individual organs has been well demonstrated using the electron spin resonance (ESR) technique.^(1–5)^ Previous studies have demonstrated that ESR in combination with 3-carbamoyl-2,2,5,5-tetramethylpyrrolidine-1-oxyl (carbamoyl-PROXYL) is sensitive for evaluation of *in vivo* organ redox activity. Carbamoyl-PROXYL is reduced to the corresponding hydroxylamine, resulting in disappearance of the ESR signal of carbamoyl-PROXYL (Fig. 1).^(2)^ Sulfhydryl (SH) compounds including reduced glutathione have been implicated in the process of carbamoyl-PROXYL reduction.^(4,5)^ ESR in combination with carbamoyl-PROXYL is useful for evaluating the tissue defense system against oxidative stress, particularly before the onset of obvious hepatic injury or during the mucosal regeneration process.^(4,5)^ However, it is difficult to obtain precise anatomical information from ESR imaging, and so far the mouse represents the maximum animal size to which the technique is applicable because of limitations on the size of the loop-cap resonator and the penetration depth of microwaves, thus preventing its clinical application.

Magnetic resonance imaging (MRI) is a useful diagnostic tool with excellent spatial resolution. It also provides detailed anatomical information on the whole body in a single examination. Carbamoyl-PROXYL is known to be T1-sensitive and has been used as a clinical contrast agent for MRI.^(6)^ Because of the rapid development and evolution of MRI systems, we expect that MRI will allow high-resolution monitoring of nitroxide dynamics in organs while simultaneously providing detailed anatomical information on redox activity. However, little attempt has been made to apply MRI clinically for spatiotemporal evaluation of redox activity, for example using animals larger than mice or developing MRI instruments that would be clinically practicable. *In vivo* MRI for visualization of redox status would provide clinically useful information for analyzing pathophysiological conditions and subsequent organ injury. The purpose of the present study was to establish a method for *in vivo* spatiotemporal visualization of redox status by high-resolution whole-body MRI using carbamoyl-PROXYL as a probe and to develop the technology for future clinical application.

## Materials and Methods

### Chemicals

Carbamoyl-PROXYL and ascorbic acid were purchased from Sigma-Aldrich Chem. Co. (St. Louis, MO). Distilled, deionized water was used for phantom experiments. For *in vivo* imaging of animals, carbamoyl-PROXYL was dissolved in isotonic sodium chloride solution for intravenous injection.

### Animal studies

Five male Wistar rats (Japan SLC Inc., Shizuoka, Japan) were used in the present study. The rats were housed in climate-controlled stainless steel cages with a standard pellet diet and tap water *ad libitum* and maintained on a 12-h light-dark cycle. The rats were 9 weeks of age weighing 210–250 g at the time of imaging. The care, maintenance, and all *in vivo* experiments with the animals were carried out in compliance with the “Guide for the Care and Use of Laboratory Animals” published by the National Institutes of Health and the Guidelines of the Animal Investigation Committee of the University. For *in vivo* imaging studies, each rat was anesthetized with an intraperitoneal injection of mixed anesthetic agents comprising 0.3 mg/kg medetomidine, 4.0 mg/kg midazolam, and 5.0 mg/kg butorphanol.

### Imaging protocol

All MRI measurements were performed using at an Achieva 3.0-Tesla Quasar Dual (Philips, Amsterdam, The Netherlands). Imaging parameters were as follows: T1-weighted incoherent gradient-echo sequence, repetition time = 3.6 ms, echo time = 1.76 ms, flip angle = 10 degrees, field of view = 320 mm × 192 mm, number of averages = 1, scan time = 25.4 s, matrix = 480 × 480, slice thickness = 1.2 mm, and number of slices = 140. Coronal slices with a 0.67 × 0.67 × 1.2 mm^3^ nominal voxel resolution were selected. The MRI data were analyzed using Extended MR WorkSpace 2.6.3.5 (Philips, Amsterdam, The Netherlands).

### Phantom experiments

Eight test tubes (internal diameter 1.27 cm) for use as phantoms were set in a polypropylene box filled with water. Each cylindrical phantom containing 10 ml of 0–80 mM carbamoyl-PROXYL solution was evaluated by MRI for determining the optimal concentration for intravenous injection. In addition, ascorbic acid solution was prepared as a reductant with which to reduce carbamoyl-PROXYL to the corresponding hydroxylamine. Solutions of 4 mM carbamoyl-PROXYL and 10 mM ascorbic acid were prepared in deionized water. Five milliliters of ascorbic acid solution was added to the same volume of carbamoyl-PROXYL. T1-weighted imaging was started 5 min after the reaction of carbamoyl-PROXYL with ascorbic acid, and repeated at 1-min intervals for up to 30 min.

### *In vivo* MRI experiments

The rats were anesthetized using mixed anesthetic agents and placed inside the MRI system with a custom-made rat holder placed in an abdominal position. The tail vein was cannulated using a polyethylene tube for administration of the nitroxide probe. Before carbamoyl-PROXYL administration, control images were obtained. The carbamoyl-PROXYL was injected (0.1 mmol/g body weight) with the aim of maintaining the blood carbamoyl-PROXYL concentration higher than 40 mM. Immediately after the carbamoyl-PROXYL injection, T1-weighted imaging was performed continuously for up to 20 min and repeated at approximately 2–3-min intervals. To analyze the MRI data, regions of interest (ROIs) were selected: (i) the heart, (ii) the liver, (iii) the kidney, and (iv) the small intestine. The time course of the signal intensity of these four organs was each measured separately. Rats injected with isotonic sodium chloride solution served as negative controls.

### Statistical analysis

Statistical analysis was performed using the R statistical package ver. 3.1.0 (R Core team 2014. R: A language and environment for statistical computing. R Foundation for Statistical Computing, Vienna, Austria, http://www.R-project.org/). All data were expressed as means ± SD unless otherwise indicated. The signal intensity of MRI measurements was taken as the logarithm, and subjected to linear regression analysis. Statistical comparisons among all groups were performed using one-way analysis of variance (ANOVA) followed by Tukey’s honestly significant difference test. Differences at *p*<0.05 were considered to be statistically significant.

## Results

**Fig. 2:**
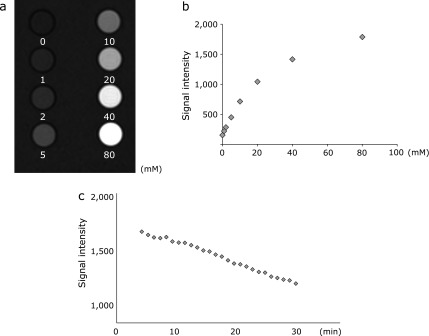
Phantom images obtained by T1-weighted MRI. (a) Phantom objects containing several different concentrations of carbamoyl-PROXYL (0–80 mM). (b) The carbamoyl-PROXYL-enhanced MRI signal was dose-dependently detectable. (c) Decay process seen in a cylindrical phantom consisting of a mixture of carbamoyl-PROXYL and ascorbic acid.

Figure 2 shows the T1-weighted MRI images of the phantoms containing several different concentrations of carbamoyl-PROXYL. The carbamoyl-PROXYL-enhanced MRI signal showed a linear correlation with the carbamoyl-PROXYL concentration up to 80 mM (Fig. 2a and b). The tube containing 5 mM carbamoyl-PROXYL was successfully imaged. T1-weighted MRI has the same sensitivity for imaging carbamoyl-PROXYL distribution as an L-band ESR instrument.

Nitroxides including carbamoyl-PROXYL are redox-sensitive probes that are reduced to the corresponding hydroxylamines (Fig. 1). To check whether the observed T1-weighted MRI values were truly ascribable to carbamoyl-PROXYL, we mixed carbamoyl-PROXYL with ascorbic acid, a strong reductant, and examined the decay of the T1-weighted MRI signal of carbamoyl-PROXYL. As shown in Fig. 2c, the MRI signal intensity decreased dramatically after addition of ascorbic acid, whereas the control without ascorbic acid showed no change (data not shown). Therefore, the observed T1-weighted MRI signal originated from the carbamoyl-PROXYL.

**Fig. 3:**
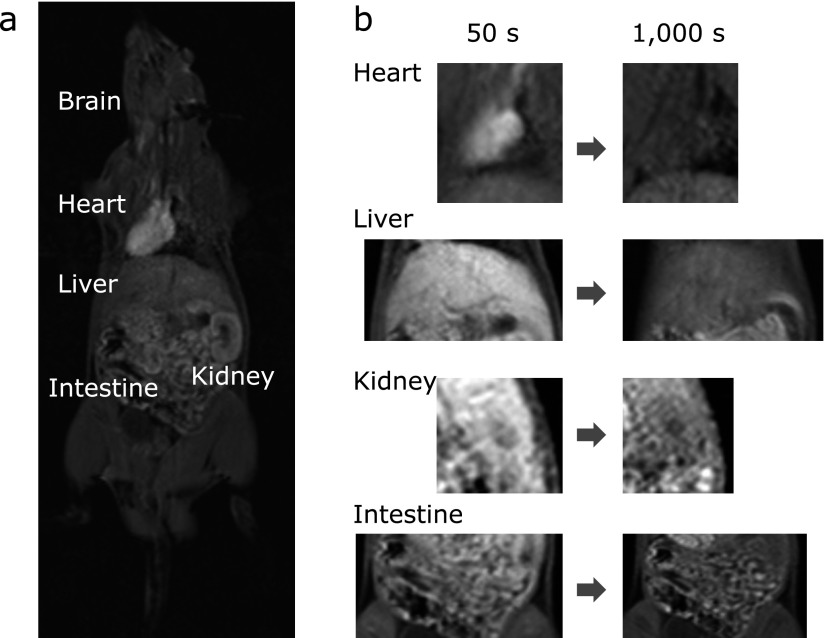
Typical T1-weighted MRI images after intravenous injection of carbamoyl-PROXYL, and a time course study. (a) Coronal sectional images of the rat whole body. (b) T1-weighted images of rat heart, liver, kidney, and intestine. Reduction of carbamoyl-PROXYL was imaged clearly by T1-weighted MRI.

Unlike ESR imaging, MRI provides images with useful spatial and temporal resolution. Our previous studies and others had shown that nitroxides including carbamoyl-PROXYL are redox-sensitive probes, reacting with reducing species such as SH compounds in the body, and thereby easily losing its ESR signal through a single electron reduction. Figure 3 shows typical *in vivo* T1-weighted MRI images after intravenous injection of carbamoyl-PROXYL. Coronal sections of the rat whole body were imaged clearly (Fig. 3a). The administered carbamoyl-PROXYL was carried via capillaries, and high levels of T1-weighted MRI enhancement were seen in the heart, liver, kidney, and intestines. Because of the blood-brain barrier, the distribution of carbamoyl-PROXYL in the brain was less than that in the other organs (Fig. 3a). Fifty seconds after carbamoyl-PROXYL injection, the heart, liver, kidney, and intestines were clearly visualized with high signal intensity. The signal in these organs declined gradually and reached the background level at 1,000 s (Fig. 3b).

**Fig. 4:**
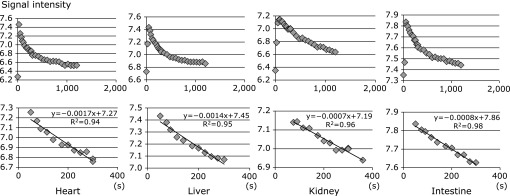
Process of decay of the MRI signal intensity in the heart, liver, kidney and intestine. The signal intensity of MRI measurements was taken as the logarithm, and linear regression analysis was performed. The organ-specific reduction rate of carbamoyl-PROXYL was calculated in each case.

From the results of the present study, we expected that T1-weighted MRI in combination with carbamoyl-PROXYL would be capable of evaluating the *in vivo* redox status of organs systematically. T1-weighted MRI sensitive reduction of carbamoyl-PROXYL was compared at the same level at intervals of 20–30 s after carbamoyl-PROXYL injection. Four organs—the heart, liver, kidney, and intestine—were selected for evaluation of *in vivo* redox status. ROIs were selected and signal intensity of the carbamoyl-PROXYL-enhanced MRI signal was analyzed quantitatively. A T1-weighted MRI time course study demonstrated that high-intensity areas of carbamoyl-PROXYL peaked and declined gradually, as shown in Fig. 4. The initial enhancement of the MRI signal might have been due to the presence of carbamoyl-PROXYL in the blood and extracellular space of organs, whereas the decrease was due to its reduction to the corresponding hydroxylamine by the organs. As shown in Fig. 4, inter-organ differences in the carbamoyl-PROXYL reduction rate in healthy rats were observed, suggesting that the redox activity differed among the organs.

**Fig. 5:**
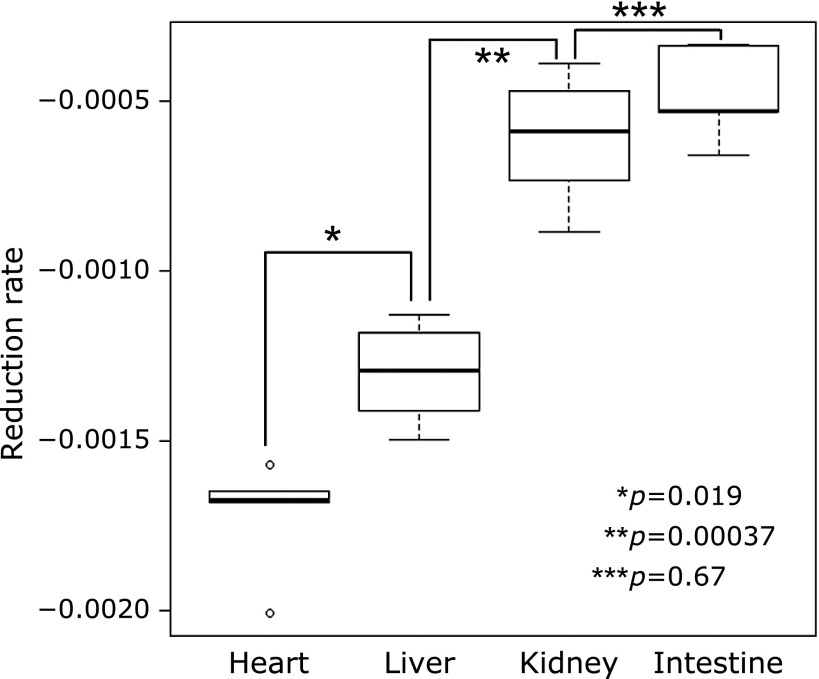
Analysis of the organ-specific reduction rate of carbamoyl-PROXYL. The reduction rate was fastest in the heart, followed in order by the liver, kidney, and intestine. There were significant differences between the heart and liver, and between the liver and kidney. The reduction rate was slow in the intestine.

Because the signal intensity of carbamoyl-PROXYL was linear for several min after the peak, we calculated the organ-specific reduction rate of carbamoyl-PROXYL. Among the four organs studied, reduction rate was most rapid in the heart, followed in order by the liver, kidney, and intestine (Fig. 5). Statistical analysis showed that there were significant differences in reduction rate between the heart and liver, and between the liver and kidney (*p*<0.02 and *p*<0.0005, respectively). Reduction rate was slow in the intestine. Because carbamoyl-PROXYL reduction depends upon the availability of reductants, the observed inter-organ differences are thought to reflect corresponding differences in redox activity.

## Discussion

Both ESR and MRI are resonance techniques that record spin transitions when a system in the magnetic field is exposed to adequate electromagnetic irradiation. The most important difference between ESR and MRI is the species that they detect, free radicals in the former case and water protons in the latter. To date, *in vivo* detection of free radicals including the nitroxide radical has been performed using the L-band ESR system.^(1–5)^ Although the L-band ESR system has the advantage of high specificity and sensitivity, the L-band ESR instrument does not have very good spatial resolution and the penetration depth of the microwave radiation is limited, thus preventing its clinical application. T1-relaxation of protons can be affected by paramagnetic nitroxide electron spin. As a result, the nitroxide radical has T1-shortening properties and enhances the T1-weighted MRI signal. Nitroxide radicals can be seen indirectly through their effect on the relaxation of water protons. In the early 80s, nitroxides were studied as clinical contrast agents for MRI.^(7)^ As MRI systems have been evolving rapidly, we expected that MRI would allow high-resolution monitoring of nitroxide dynamics in organs while simultaneously providing detailed anatomical information. Our basic experiment showed that the carbamoyl-PROXYL-enhanced MRI signal was linearly correlated with the carbamoyl-PROXYL concentration up to 80 mM, and even the tube containing a low concentration (1 mM) was readily identifiable in the phantom. Moreover, ascorbic acid, a potent reductant, reduced the T1-weighted MRI signal. We considered that T1-weighted MRI would be able to specifically monitor *in vivo* nitroxide radical dynamics within the body. The present *in vivo* study demonstrated an inter-organ difference in the time taken for the carbamoyl-PROXYL signal to peak after intravenous injection, and also in the signal decay speed after the peak. Although the toxicity of nitroxide radicals, including carbamoyl-PROXYL, is a concern, we consider that the present study represents a milestone in the development of techniques for prevention and diagnosis of human diseases whose onset and progression are related to oxidative stress.

Carbamoyl-PROXYL was reduced to the corresponding hydroxylamine, resulting in disappearance of the ESR signal of carbamoyl-PROXYL (Fig. 1).^(2)^ SH compounds including reduced glutathione have been implicated in the process of carbamoyl-PROXYL reduction.^(4,5)^ The ESR instrument in combination with carbamoyl-PROXYL is useful for evaluating the tissue defense system against oxidative stress, particularly before the onset of obvious hepatic injury or during the mucosal regeneration process.^(4,5)^ Other research groups have considered that the bio-reduction of nitroxides could be an effective probe reflecting redox status.^(6,8)^ Matsumoto *et al.*^(9)^ performed comparative studies of the process of carbamoyl-PROXYL reduction, an indicator of tissue redox status, in tumors implanted into the hind limbs of mice using electron paramagnetic resonance (EPR; synonymous with ESR) spectroscopy and MRI. They obtained clear and important proof that redox status determined by EPR and MRI showed a similar trend. Although we did not measure the contents of antioxidants including SH compounds, the rate of carbamoyl-PROXYL reduction measured by T1-weighted MRI was considered to reflect redox status, being similar to that determined by EPR spectroscopy. In the present study, we clarified carbamoyl-PROXYL accumulation and reduction process in whole organs with high anatomical resolution. Because rat is bigger than mouse in size, we performed ROI analysis with large areas and quantified the T1-weighted signal originated from carbamoyl-PROXYL. The present method is superior to the previous method in its quantification. Signal intensity of carbamoyl-PROXYL was linear during several min after the peak, we calculated the kinetic clearance rate of organs. The reduction of nitroxide take place in (1) the bloodstream, (2) the extracellular space of tissue, and (3) the cells in the tissue. The kinetic clearance rate can give an estimate of the level of SH compounds in organs using the ESR instrument.^(4,5)^ The present study showed that reduction rates, i.e., redox activities, differed among various organs. In fact in normal rats, the defense system against oxidative stress has been reported to differ similarly among organs.^(10)^ Our data accord with that report, and suggest that the present approach could be a convenient method for evaluation of whole-organ redox activity at the same time. The present study clearly revealed that analysis of carbamoyl-PROXYL dynamics using 3-T clinical MRI yields a sensitivity comparable to L-band ESR. Moreover, MRI in combination with carbamoyl-PROXYL shows excellent spatiotemporal resolution and has the potential for clinical application for evaluation of whole-organ redox activity.

Because nitroxide radicals including carbamoyl-PROXYL can yield a T1-weighted signal in MRI, they were once used as T1-enhancing MRI contrast agents during the early development of MRI.^(7)^ However, their bio-reduction and rapid clearance from the body prevented their clinical application. The rapid progress of MR scanning techniques such as the spoiled gradient echo method for rapid imaging has made it possible to monitor and image the distribution of carbamoyl-PROXYL in various organs in real time. Currently, three different techniques, ESR-MRI, Overhauser MRI, and MRI are available for redox imaging using nitroxide radicals.^(11,12)^ Using these techniques, the processes of reduction of nitroxide radicals in the brains and implanted tumors of mice have been studied. These MRI techniques have been reported to be quite useful for *in vivo* evaluation of redox activity, but at the time were under development as experimental techniques and not considered ready for clinical application. We considered that 3-T clinical MRI would be practicable if we conducted animal experiments with a view to their future application to analysis of human diseases. Use of rats allowed a large ROI to be selected, thereby increasing the reliability of the data considerably. Even though carbamoyl-PROXYL shows less toxicity, the safety of nitroxide radicals including carbamoyl-PROXYL will need to be confirmed before they are applied to humans.

In conclusion, the present study is the first to have examined redox activity in various organs *in vivo* simultaneously, and is regarded as translational research for future clinical application.
